# Supplementary material for: Succession comprises a sequence of threshold-induced community assembly processes towards multidiversity
Source: Commun Biol. 2022 May 6;5:424. doi: 10.1038/s42003-022-03372-2 (PMC9076875; doi:10.1038/s42003-022-03372-2)
Supplement: Supplementary file 1 — Supplementary Information [file 42003_2022_3372_MOESM1_ESM.pdf]

Supporting information for:

Succession comprises a sequence of threshold-induced community assembly processes towards multidiversity

Maximilian Hanusch[1], Xie He[1], Victoria Ruiz-Hernández[1], Robert R. Junker[1,2*]

[1]Department of Environment and Biodiversity, Paris Lodron University Salzburg, 5020 Salzburg, Austria

[2]Evolutionary Ecology of Plants, Department of Biology, Philipps-University Marburg, 35043 Marburg, Germany

*Corresponding author: robert.junker@uni-marburg.de

Supplementary table 1: Summary of the Leave-One-Out cross validation for breaking-point model comparison and Bayes Factor (BF) analysis for testing of exact location of the breaking point. The best supported model is chosen by the difference in Estimated Log Predictive Density (ELPD) and Standard Error of the model. The most likely changepoint (cp) is chosen according to BF. Higher BF-values indicate a higher probability of the tested hypothesis about the cp. Asterisks mark the most probable hypothesis.

Model	ELPD diff	SE diff
m4	0.0	0.0
m3	-1.5	1.5
m2	-7.2	3.3
m1	-13.9	5.2
Hypothesis	BF	
cp = 42	6.62	
cp = 43	6.85	
cp = 44*	6.87*	
cp = 45	6.45	
cp = 46	5.75	
cp < 43	1.05	
cp < 44	1.36	
cp < 45*	1.77*	
cp > 43*	0.94*	
cp > 44	0.73	
cp > 45	0.56	

Supplementary table 2: Summary of the test for spatial autocorrelation in the residuals of the dependent variables in the linear models and path analysis. Spatial autocorrelation was tested for neighbor lists of 5 m and 10 m distance classes.

Distance class 5 m				
Model	Successional stage	Variable	Moran´s I	p-value
Linear model	early	Multidiversity	-0.00	0.29
Linear model	late	Multidiversity	0.03	0.27
Path analysis	early	Vasc. Plant diversity	0.01	0.28
Path analysis	early	Bryophyte diversity	0.05	0.26
Path analysis	early	Bacterial diversity	0.04	0.26
Path analysis	early	Fungal diversity	0.03	0.27
Path analysis	early	Invertebrate diversity	-0.02	0.30
Path analysis	early	Multidiversity	0.01	0.29
Path analysis	late	Vasc. Plant diversity	0.00	0.28
Path analysis	late	Bryophyte diversity	0.05	0.26
Path analysis	late	Bacterial diversity	0.04	0.26
Path analysis	late	Fungal diversity	0.00	0.26
Path analysis	late	Invertebrate diversity	0.01	0.28
Path analysis	late	Multidiversity	0.02	0.27
Distance class 10 m				
Model residual	successional stage	variable	Moran´s I	p-value
Linear model	early	Multidiversity	0.05	0.33
Linear model	late	Multidiversity	0.14	0.20
Path analysis	early	Vasc. Plant diversity	0.10	0.28
Path analysis	early	Bryophyte diversity	-0.07	0.53
Path analysis	early	Bacterial diversity	0.06	0.32
Path analysis	early	Fungal diversity	-0.09	0.56
Path analysis	early	Invertebrate diversity	0.03	0.37
Path analysis	early	Multidiversity	0.16	0.20
Path analysis	late	Vasc. Plant diversity	0.13	0.22
Path analysis	late	Bryophyte diversity	0.08	0.3
Path analysis	late	Bacterial diversity	0.17	0.16
Path analysis	late	Fungal diversity	0.07	0.32
Path analysis	late	Invertebrate diversity	-0.01	0.47
Path analysis	late	Multidiversity	0.13	0.21
