# Supplementary material for: Succession comprises a sequence of threshold-induced community assembly processes towards multidiversity
Source: Commun Biol. 2022 May 6;5:424. doi: 10.1038/s42003-022-03372-2 (PMC9076875; doi:10.1038/s42003-022-03372-2)
Supplement: Supplementary file 2 — Description of Additional Supplementary Files [file 42003_2022_3372_MOESM2_ESM.pdf]

Description of Additional Supplementary Files

File name: Supplementary Data 1
Description: Summary of the path analysis of the effects of time since deglaciation, temperature, pH-value and soil nutrients on organismal groups and multidiversity (model not used).

File name: Supplementary Data 2
Description: Summary of the path analysis of the effects of time since deglaciation, temperature and pH-value on organismal groups and multidiversity (model not used).

File name: Supplementary Data 3
Description: Summary of the path analysis of the effects of time since deglaciation, temperature and soil nutrients on organismal groups and multidiversity (model not used).

File name: Supplementary Data 4
Description: Summary of the path analysis of the effects of time since deglaciation and temperature on organismal groups and multidiversity (final model).
