# Supplementary material for: Succession comprises a sequence of threshold-induced community assembly processes towards multidiversity
Source: Commun Biol. 2022 May 6;5:424. doi: 10.1038/s42003-022-03372-2 (PMC9076875; doi:10.1038/s42003-022-03372-2)
Supplement: Supplementary file 7 — Reporting Summary [file 42003_2022_3372_MOESM7_ESM.pdf]

nature portfolio

Corresponding author(s): Robert R. Junker robert.junker@uni-marburg.de

Last updated by author(s): Feb 25, 2022

Reporting Summary

Nature Portfolio wishes to improve the reproducibility of the work that we publish. This form provides structure for consistency and transparency in reporting. For further information on Nature Portfolio policies, see our Editorial Policies and the Editorial Policy Checklist.

Statistics

For all statistical analyses, confirm that the following items are present in the figure legend, table legend, main text, or Methods section.

n/a	Confirmed	
☐	☒	The exact sample size (n) for each experimental group/condition, given as a discrete number and unit of measurement
☐	☒	A statement on whether measurements were taken from distinct samples or whether the same sample was measured repeatedly
☐	☒	The statistical test(s) used AND whether they are one- or two-sided<br>*Only common tests should be described solely by name; describe more complex techniques in the Methods section.*
☐	☒	A description of all covariates tested
☐	☒	A description of any assumptions or corrections, such as tests of normality and adjustment for multiple comparisons
☐	☒	A full description of the statistical parameters including central tendency (e.g. means) or other basic estimates (e.g. regression coefficient) AND variation (e.g. standard deviation) or associated estimates of uncertainty (e.g. confidence intervals)
☐	☒	For null hypothesis testing, the test statistic (e.g. F, t, r) with confidence intervals, effect sizes, degrees of freedom and P value noted<br>*Give P values as exact values whenever suitable.*
☐	☒	For Bayesian analysis, information on the choice of priors and Markov chain Monte Carlo settings
☒	☐	For hierarchical and complex designs, identification of the appropriate level for tests and full reporting of outcomes
☐	☒	Estimates of effect sizes (e.g. Cohen's d, Pearson's r), indicating how they were calculated

Our web collection on statistics for biologists contains articles on many of the points above.

Software and code

Policy information about availability of computer code

Data collection	Q-GIS 3.4 was used for GIS-based sampling site choice
Data analysis	R-4.1.0 was used for data analysis

For manuscripts utilizing custom algorithms or software that are central to the research but not yet described in published literature, software must be made available to editors and reviewers. We strongly encourage code deposition in a community repository (e.g. GitHub). See the Nature Portfolio guidelines for submitting code & software for further information.

Data

Policy information about availability of data

All manuscripts must include a data availability statement. This statement should provide the following information, where applicable:

- Accession codes, unique identifiers, or web links for publicly available datasets
- A description of any restrictions on data availability
- For clinical datasets or third party data, please ensure that the statement adheres to our policy

Raw sequences of next-generation 16S rRNA gene amplicon sequencing are available at the NCBI Sequence Read Archive (SRA) under the BioProject accession PRJNA701884 and PRJNA701890. Raw floristic and zoological data will be made available before publication.

Field-specific reporting

Please select the one below that is the best fit for your research. If you are not sure, read the appropriate sections before making your selection.

☐ Life sciences ☐ Behavioural & social sciences ☒ Ecological, evolutionary & environmental sciences

For a reference copy of the document with all sections, see nature.com/documents/nr-reporting-summary-flat.pdf

Ecological, evolutionary & environmental sciences study design

All studies must disclose on these points even when the disclosure is negative.

Study description	A study on primary succession and ecosystem complexity that is located i in the Austrian Alps. A total of n=140 sampling sites were set up along a successional gradient following glacial retreat.
Research sample	Soil inhabiting bacteria + fungi, arthropods, as well as vascular and non-vascular plants were sampled on each plot. Additionally, we sampled environmental variables such as soil temperature, soil nutrients and pH. We used GIS analysis based on historical data and geomorphological features to infer the successional age of the plots.
Sampling strategy	Data was collected on each plot. Plot size = 1square meter and plots were pre-selected by a randomized GIS-approach that allowed homogenous site distribution along the entire gradient. Detailed sampling strategy under https://doi.org/10.5194/we-20-95-2020
Data collection	Data was collected by the authors of the study. Environmental parameters and microbial data was processed in the laboratory after returning from fieldwork.
Timing and spatial scale	Data was collected throughout the vegetation periods (i.e. snow-free period, approximately June-August) during 2019 and 2020. The successional gradient covers ca. 1.7 km in length along the forefield of the Oedenwinkelkees glacier, Austria.
Data exclusions	Out of n=140 plots, in this study we used n=110 plots that all data (biotic + abiotic) was available. The subset of plots was still homogenously distributed along the gradient.
Reproducibility	All plots are GPS-located an will be re-sampled in the future through long-time ecological monitoring. Sampling strategies have been carefully noted and are available under https://doi.org/10.5194/we-20-95-2020
Randomization	Sampling along the gradient was randomized to avoid a temporal signal in the data.
Blinding	Blinding was not necessary.

Did the study involve field work? ☒ Yes ☐ No

Field work, collection and transport

Field conditions	Field work was conducted in the snow-free period (June-August). Microbial sampling was performed during dry conditions to avoid a bias of rainfall.
Location	The field site is located at approx 2100m a.sl. in an high alpine surrounding. Coord DD-format: 47.12450604493043, 12.637591314259604
Access & import/export	Sampling sites are located in the Hohe Tauern National Park, Austria. Sites were accessed by foot and samples were transported to University of Salzburg laboratories immediately after collection. Sampling permits were issued by the governing authority of the Land Salzburg (permit no. 20507-96/45/7-2019)
Disturbance	Sampling sites were not disturbed through the sampling.

Reporting for specific materials, systems and methods

We require information from authors about some types of materials, experimental systems and methods used in many studies. Here, indicate whether each material, system or method listed is relevant to your study. If you are not sure if a list item applies to your research, read the appropriate section before selecting a response.

Materials & experimental systems

n/a	Involved in the study	
☒	☐	Antibodies
☒	☐	Eukaryotic cell lines
☒	☐	Palaeontology and archaeology
☐	☒	Animals and other organisms
☒	☐	Human research participants
☒	☐	Clinical data
☒	☐	Dual use research of concern

Methods

n/a	Involved in the study	
☒	☐	ChIP-seq
☒	☐	Flow cytometry
☒	☐	MRI-based neuroimaging

Animals and other organisms

Policy information about studies involving animals; ARRIVE guidelines recommended for reporting animal research

Laboratory animals	The study did not involve laboratory animals.
Wild animals	Arthropods were caught using pitfall-traps filled with a mixture of ethylene-glycole and water.
Field-collected samples	Caught arthropods are stored in ethanol in the zoological collection of The University of Salzburg
Ethics oversight	The governing authorities of the Land Salzburg approved the collection of arthropods within the National Park.

Note that full information on the approval of the study protocol must also be provided in the manuscript.
